# Rivaroxaban versus low-molecular-weight heparin for venous thromboembolism prophylaxis: a systematic review of efficacy, safety, and cost-effectiveness

**DOI:** 10.3389/fphar.2025.1652779

**Published:** 2025-09-25

**Authors:** Bin Li, Ling Zhao

**Affiliations:** ^1^ Department of Pharmacy, The People’s Hospital Medical Group of Xiangzhou, Zhuhai, Guangdong, China; ^2^ Department of Intensive Care Unit, The People’s Hospital Medical Group of Xiangzhou, Zhuhai, Guangdong, China

**Keywords:** rivaroxaban, low-molecular-weight heparin, venous thromboembolism, anticoagulation, cost-effectiveness

## Abstract

Hospitalization significantly elevates venous thromboembolism (VTE) risk, yet optimal prophylactic anticoagulation remains debated. While low-molecular-weight heparin (LMWH) has long been the standard, direct oral anticoagulants (DOACs) like rivaroxaban offer oral administration without routine monitoring. We systematically reviewed 24 studies identified through PubMed and Web of Science (2010–2024), using predefined inclusion and exclusion criteria. Studies were screened, duplicates removed, and data extracted on efficacy, safety, cost-effectiveness, and patient-centered outcomes. High-risk subgroups, such as those with cancer or renal impairment were a focus of analysis. Efficacy: Rivaroxaban demonstrated non-inferiority to LMWH in VTE prevention, with superior postoperative thromboprophylaxis in orthopedic patients. Safety: Minor bleeding incidence was marginally higher with rivaroxaban, but major bleeding risk was comparable. Economics: Rivaroxaban reduced costs by approximately $1,200 per VTE patient, particularly in cancer-related cases. Rivaroxaban provides equally effective VTE prophylaxis with enhanced practicality (oral administration, no monitoring) and significant healthcare savings. Therapy selection should prioritize renal function, cancer status, and bleeding risk. Future research must address DOAC safety in severe renal/hepatic impairment and optimize reversal-agent accessibility.

## 1 Introduction

Venous thromboembolism (VTE), encompassing deep vein thrombosis (DVT) and pulmonary embolism (PE), is a leading cause of mortality and morbidity in the hospital population. Hospitalization independently raises thrombosis risk by causing immobility, systemic inflammation, and comorbid medical illness. Prophylactic anticoagulation is therefore warranted to reduce preventable VTE-related death. Low-molecular-weight heparin (LMWH) has been the treatment standard with an excellent evidence base and consistent efficacy at infrequent laboratory monitoring for decades. However, limitations such as subcutaneous administration, pain for the patient, and the requirement of dose adjustment in renal failure have prompted the search for alternatives. Direct oral anticoagulants (DOACs) such as rivaroxaban represent an essential advance in thromboprophylaxis. With oral dosing, fixed dosing regimens, and no requirement for routine monitoring, DOACs have been more commonly used in inpatient and outpatient settings. Convenience is particularly relevant to care transitions and extended prophylaxis, where injections might compromise compliance. Nice as these are, questions of relative safety, effectiveness in high-risk subgroups, and cost-effectiveness in different healthcare systems persist.

This review systematically compares rivaroxaban and LMWH for VTE prophylaxis in medical inpatients based on efficacy, safety, cost-effectiveness, and patient-centered outcomes, focusing on high-risk subgroups such as cancer and renal impairment patients. The pharmacological rationale for these agents differs considerably: rivaroxaban inhibits factor Xa directly, and LMWH enhances antithrombin III activity. These mechanisms are described in greater detail in Section 3 of this review. Of most tremendous clinical significance is how such differences endure in affecting patient outcomes. Issues of safety, most significantly risk of bleeding, remain uppermost in the decision-making process. Data from retrospective reports provide inconclusive findings. For instance Wu et al. (2024a) showed in a study of 180 hospitalized cancer patients that rivaroxaban was associated with higher major bleeding than LMWH (8.9% vs. 2.2%; p < 0.05). Conversely Deitelzweig et al. (2015), in a real-world cohort of 512 DVT patients, found no considerable difference in hospitalization due to major bleeding between rivaroxaban and LMWH (1.2% vs. 1.5%; p > 0.05). Evidence such as this helps to emphasize systematic synthesis to firm up the weighing of risk and benefit. Clinical effectiveness evidence also indicates overall comparability, with observational studies and meta-analyses indicating similar prevention of recurrent VTE, particularly in well-defined high-risk conditions such as cancer patients (Bozzato et al., 2013). Rivaroxaban has been demonstrated to be non-inferior to LMWH in many large-scale trials, and in specific subsets, such as patients undergoing orthopedic surgery with postoperative prophylaxis, has been shown to have superior efficacy in thromboprophylaxis. This efficacy and oral delivery make rivaroxaban an increasingly realistic alternative in clinical practice.

Economic considerations influence the choice of anticoagulants. Cost-effectiveness models suggest that rivaroxaban has the potential to reduce healthcare expenditures by preventing injection-related use of healthcare resources, reducing monitoring, and improving patient compliance Deitelzweig et al. (2016) demonstrated that rivaroxaban treatment of outpatients with DVT had mean healthcare costs of $4,789 per patient at 6 months compared to LMWH. Similarly Bamber et al. (2015), with a Markov model in the UK National Health Service, estimated lifetime savings of £433 per patient when rivaroxaban was utilized compared to LMWH. These findings highlight rivaroxaban’s potential for decreasing healthcare resource use without compromising efficacy, although these savings will vary by healthcare system and patient subgroup.

Aside from expense, patient self-report of convenience, quality of life, and treatment satisfaction are increasingly essential outcomes. Oral administration of rivaroxaban also spares patients injections and the accompanying pain, potentially promoting adherence and reducing treatment discontinuation. These benefits must be weighed against residual uncertainty, particularly in trial-underrepresented groups like elderly individuals, patients with severe renal or hepatic impairment, and individuals with complex multimorbidity. Information in these cohorts remains limited, and proper caution should be used when extrapolating information. Finally, the future leads for anticoagulation (Cohen et al., 2024). Availability and access to reversal drugs for rivaroxaban remain limited, and this can complicate management with significant bleeding or urgent surgery. Moreover, pharmacogenomic data and novel biomarkers may continue to tailor patient selection in the next few years, permitting more precise and individualized prophylaxis strategies.

Briefly, VTE prevention continues to be a high priority for inpatients. While LMWH is still the reliable standard, adding rivaroxaban has offered a convenient and cost-effective alternative. Current evidence supports equal efficacy and similar safety, though data from specific subgroups reflect some uncertainty. By systematically synthesizing available evidence, this review aims to clarify the comparative merit of rivaroxaban and LMWH, advise clinicians and policymakers, and define future research agendas.

## 2 Methods

In the interest of methodological transparency and reproducibility, the methods used in this systematic review are explained below in detail. The search strategy, databases, inclusion/exclusion criteria, and study selection process are described in the following section. A PRISMA flow diagram has also been included to indicate the process of the study selection (Page et al., 2021).

### 2.1 Search strategy

A literature search was carried out to identify studies that compared rivaroxaban and low-molecular-weight heparin (LMWH) to prevent and treat venous thromboembolism (VTE). The literature research was constructed to identify studies whose results evaluate efficacy, safety and economic outcomes. The following were conducted:

Keywords: Keywords and MeSH terms used in literature search were: rivaroxaban, low-molecular-weight heparin, venous thromboembolism, VTE prevention, cost-effectiveness, major bleeding, and cancer-related thrombosis.

Search Limitation: The inclusion was restricted to English-language and peer-reviewed journals. The search criteria were works published after 2010 to get an update of information and data on the anticoagulation therapy.

### 2.2 Databases used

The following electronic databases were searched for the studies: PubMed and Web of Science. These databases were chosen to comprehensively cover clinical trials, observational studies, and economic analyses from various disciplines.

### 2.3 Inclusion and exclusion criteria

To be included in this review, studies had to meet the following criteria: Population: Adult (≥18 years) patients with diagnosed venous thromboembolism (VTE) or at high risk for VTE (cancer patients and orthopedic surgical patients). Intervention: Rivaroxaban use compared with LMWH for VTE prevention or treatment. Outcomes: Reporting primary outcomes of VTE recurrence, major bleeding, all-cause mortality, and cost-effectiveness (QALY or total healthcare expenditure). Study Design: Randomized controlled trials (RCTs), cohort studies, case-control studies, and economic analyses.

Exclusion criteria were: Non-peer-reviewed articles, case reports, or editorials. Studies in pediatric populations (<18 years). Studies with sparse data on comparing rivaroxaban and LMWH.

### 2.4 Data extraction and study selection

Data from the eligible studies were extracted independently by two reviewers to ensure consistency. The following data were extracted: Study design. Patient characteristics (age, sex, comorbidities). Type of VTE (DVT, PE). Intervention details (dosage, duration of treatment). Outcomes: VTE recurrence, major bleeding, mortality, and economic consequences. Disagreements were resolved by consensus or consulting a third reviewer.

### 2.5 PRISMA flow diagram

**FIGURE 1:**
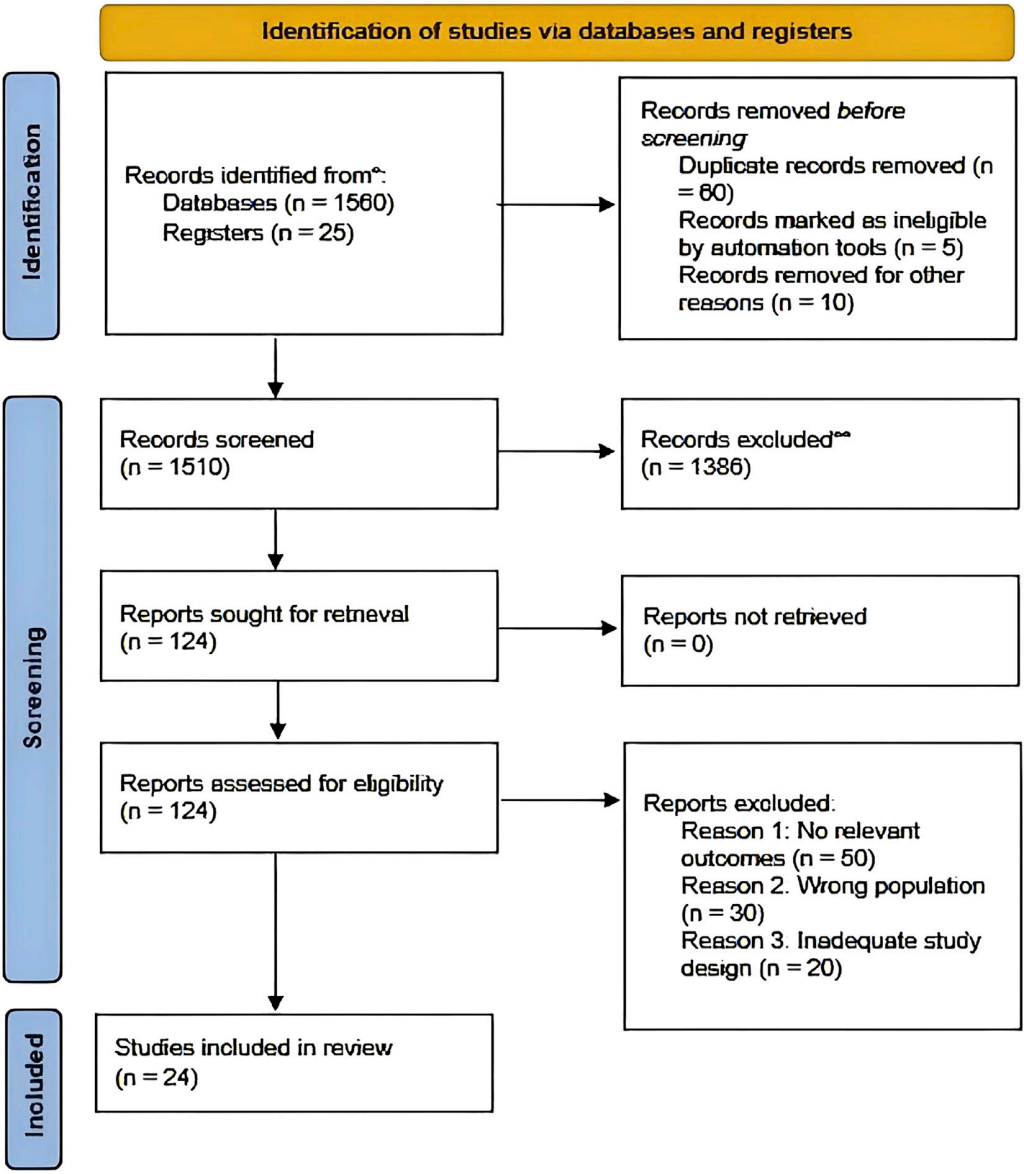
Prisma flow diagram of study selection process.

For transparency and methodological rigor, a PRISMA flow diagram was created to display the study selection process (Figure 1). The diagram shows the number of records identified, screened, eligible studies, and those ultimately included in the review.

### 2.6 Limitations

Although every effort was made to identify all studies relevant to this, the following limitations exist:Heterogeneity of populations, interventions, and outcome reporting studies. Real-world evidence included in the review has the possibility of introducing bias by patient selection and lack of randomization. Publication bias may have influenced the results, as studies with negative or null results are less likely to be published.

### 2.7 Summary

This systematic review provides a comprehensive synthesis of the evidence comparing rivaroxaban and LMWH for the management of VTE. It will include high-quality studies, such as those derived from the EINSTEIN and SEER-Medicare databases, allowing for the possibility of making definitive conclusions regarding efficacy, safety, and cost-effectiveness. The PRISMA flow diagram and data extraction procedures ensure transparency, and the results will guide clinicians and policymakers in their decision-making.

## 3 Overview of anticoagulant therapies for venous thromboembolism

Anticoagulant therapy forms the basis of VTE prevention and treatment, which predominantly appears as pulmonary embolism (PE) and deep vein thrombosis (DVT). Two agents that dominate the clinic involve using low-molecular-weight heparins (LMWHs) and rivaroxaban, a direct oral anticoagulant (DOAC). They both exhibit efficacy in preventing and treating VTE, yet they differ in their pharmacology, administration, and safety, which limits their clinical practice.

The increasing popularity of the drug Rivaroxaban is due to the predictability of the pharmacokinetics, the orality of the drug, and the lack of any extensive monitoring requirements. UFH, its derivatives, LMWHs, which are administered subcutaneously, are still utilized often because of the reliability, safety, and reduction in the risk of HIT. Both drugs can be used in a majority of populations, such as in cancer-related thrombosis, whereby extensive targeted medication is becoming of significance. In another study by Coleman et al. (2022), the study was retrospective with a cohort study of 602 hospitalized patients with cancer, and no significant difference was identified between rivaroxaban and LMWH regarding VTE prophylaxis (OR = 0.85, 95% CI: 0.39–1.87, p = 0.688). The results show that the re-optimal drug should consider comorbidities, renal status, cancer status, and compliance in improving the outcome.

### 3.1 Mechanisms of action of rivaroxaban

Rivaroxaban is a direct oral anticoagulant (DOAC) that directly inhibits a key factor in the coagulation cascade, Factor Xa, which converts prothrombin to thrombin. Rivaroxaban inhibits thrombin generation and clot formation of fibrin as it blocks this crucial process (Hindley et al., 2023). Compared to the vitamin K antagonists like warfarin, which inhibit various clotting factors nonselectively, Rivaroxaban consistently produces an anticoagulant response.

The selectivity of this drug has been described based on a good safety profile when administered clinically. Clinical studies have shown that Rivaroxaban is effective compared to LMWH, which has a lower risk of developing an acceptable bleeding rate. Its high oral bioavailability gives it a higher plasma level with better convenience and compliance over injectable courses (Wu et al., 2024a). In addition to anticoagulation, Rivaroxaban has also been investigated to have potential anti-inflammatory effects with the prospect of seeing application in the use of Rivaroxaban in the treatment of cancer-related thrombosis, where inflammation is a primary pathognomonic driver (Coleman et al., 2022). The last properties can be another therapeutic benefit in multifactorial clinical conditions of multifactorial etiopathogenesis, when thrombogenesis is present.

### 3.2 Pharmacology of low-molecular-weight heparin

Enoxaparin and dalteparin are low-molecular-weight heparins prepared by fractionating unfractionated heparin by enzymatic or chemical means. Regarding unfractionated heparin, LMWHs have more predictable pharmacokinetics, decreased monitoring necessities, and are less likely to cause heparin-induced thrombocytopenia (Nair et al., 2020).

The mechanism behind the anticoagulant action of LMWHs has been observed because of binding to antithrombin III, increasing inhibition of Factor Xa extent, thrombin (lesser extentactor Factor IIa). Their Factor Xa selectivity differs among preparations. Enoxaparin (nominal mean molecular weight ranges of 3,800–5,000 Da) and dalteparin (ranges 5,000–6,000 Da) have ratios of anti-Xa/anti-IIa activity of 3.3–4.1:1 and 2.0–2.8:1, respectively (Merli et al., 2016). The increased anti-Xa specificity has been linked to successful prophylaxis and a marginally lower chance of bleeding complications.

In a systematic review and analysis of 25 random trials, LMWH was found to lower the chances of major bleeding by 36% as compared to unfractionated heparin (RR = 0.64, 95% CI: 0.44–0.93, p = 0.02) (Bochenek and Nizankowski, 2012). However, renal clearance is a significant limitation. LMWHs get accumulated in renal-impaired patients and need dose adjustment as creatinine clearance falls below 30 mL/min. Despite this, LMWHs have less interpatient variability than unfractionated heparin and are generally suitable for extended use in heterogeneous populations (Kohn et al., 2019).

### 3.3 Comparative safety profiles of rivaroxaban and LMWH

Safety profiles are an essential consideration in the selection of anticoagulants, particularly in high-risk individuals such as cancer patients, the elderly, or renal-impaired patients (Olie et al., 2021). The risk of bleeding and tolerance of both LMWH and rivaroxaban have been vigorously tested.

Some studies have reported a slight increase in minor bleeding with rivaroxaban compared to LMWH, but other studies have contested this. Still, risks are similar in terms of severe bleeding and the risk of all-cause mortality. Costa et al. (2020) and McBane et al. (2016), among others, discovered that rivaroxaban and LMWH had similar efficacy in preventing VTE among cancer patients, with no significant differences in serious bleeding events. These findings warrant the prospect of rivaroxaban being a safe alternative in these patient groups.

**FIGURE 2:**
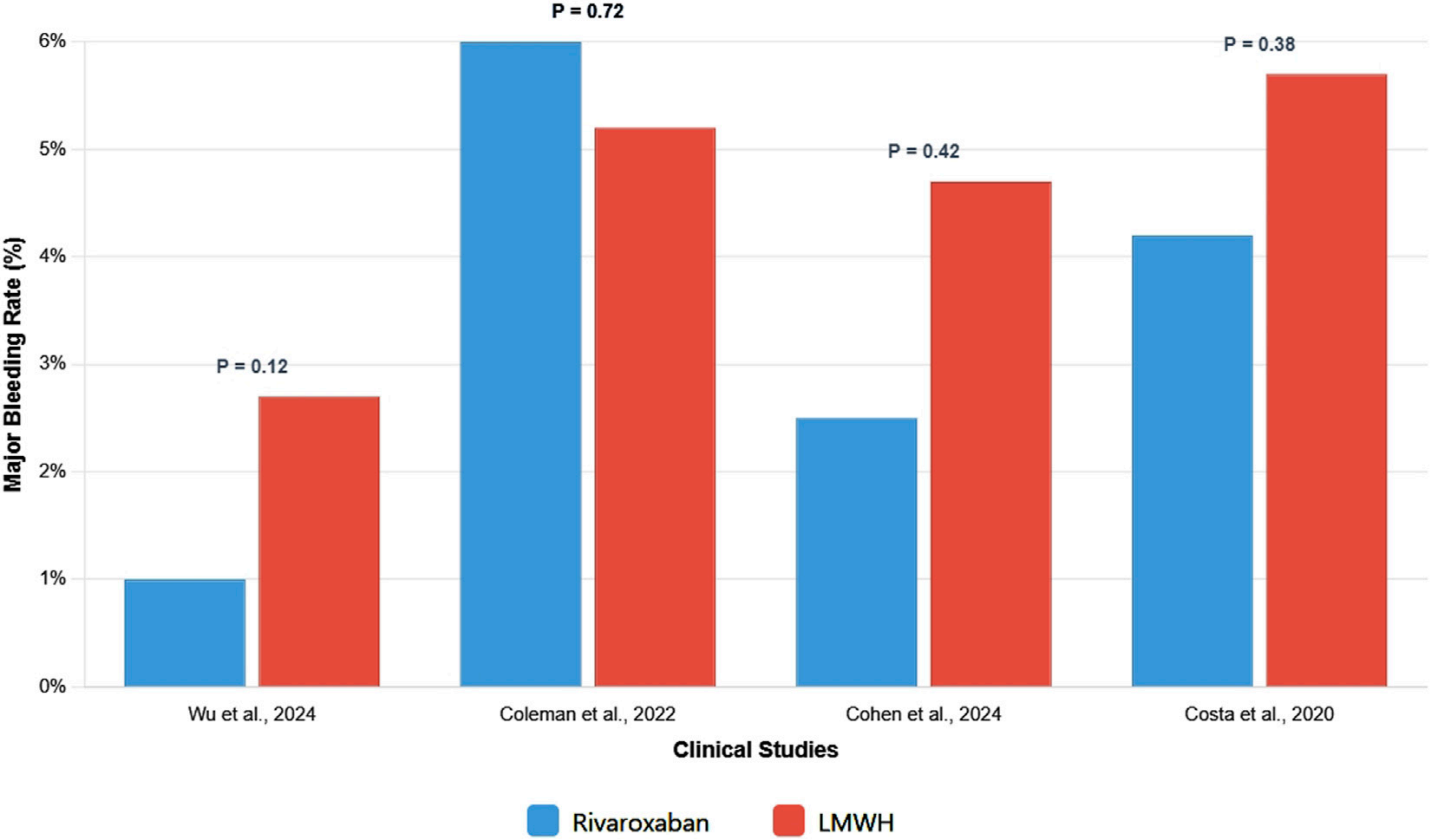
Major bleeding of Rivaroxaban versus low-molecular-weight heparin in Cancer-Associated venous thromboembolism.

LMWH safety is dependent on renal function. Reduction in dosage is needed in patients with a <30 mL/min clearance to prevent drug accumulation. Although LMWHs exhibit a high safety aspect of bleeding in most cases, renal impairment poses a significant risk (Streiff et al., 2019). Rivaroxaban, however, should only be cautiously coadministered with strong CYP3A4 and P-glycoprotein inhibitors as a result of the increased exposure of the drug and the likelihood of bleeding. Figure 2 shows relative bleeding outcomes in cancer-related thrombosis.

Drug–drug interactions further define these agents. Rivaroxaban is a substrate for CYP3A4 and P-glycoprotein. Co-administration with potent inhibitors (e.g., azole antifungals, HIV protease inhibitors) will increase plasma concentrations and risk of bleeding (Wu et al., 2024b). LMWHs undergo hepatic metabolism by depolymerization and desulfation and have fewer clinically significant drug interactions, further adding to their margin of safety in polypharmacy scenarios (van der Sangen et al., 2021). Table 1 compares rivaroxaban and LMWH regarding the most significant aspects to represent the pharmacologic and practical distinctions.

**TABLE 1:** Comparison of rivaroxaban and low-molecular-weight heparin (LMWH) in clinical and pharmacological aspects.

Feature	Rivaroxaban	LMWH
Mechanism of Action	Direct, selective inhibition of factor Xa (free and clot-associated)	Enhances antithrombin III activity; inhibits factor Xa and thrombin (IIa)
Route of Administration	Oral (fixed dosing)	Subcutaneous injection
Onset of Action	2–4 h	3–5 h
Half-life	5–13 h (varies by age/renal function)	4–7 h
Bioavailability	80%–100%	Not orally available
Clearance	Two-thirds renal, one-third hepatic (CYP3A4, CYP2J2)	Primarily renal
Monitoring Needs	None routinely; stable PK/PD	Minimal; anti-Xa monitoring in renal impairment, obesity, pregnancy
Reversal Agent	Andexanet alfa (limited availability, costly)	Protamine sulfate (partial reversal only)
Drug Interactions	Significant CYP3A4 and P-gp interactions	Few clinically significant interactions
Adverse Events	Slightly higher risk of minor bleeding; major bleeding comparable to LMWH	Generally lower risk of HIT and osteoporosis; renal impairment increases bleeding risk
Patient Convenience	Oral, avoids injections, improves adherence	Injections required; less convenient for outpatient/long-term use

The contrast between rivaroxaban and LMWH emphasizes the evolving landscape of anticoagulation. LMWH is still a safe and effective option, especially in hospitalized patients, or where drug interactions are less probable (You et al., 2025). The convenience of rivaroxaban and potential adherence benefits based on oral administration and predictable pharmacology render it an attractive option for outpatient prophylaxis and extended duration of use. Both drugs are comparable in efficacy and overall similar in safety, but their practical differences highlight the necessity for individualized decision-making (Yoon et al., 2023). In cancer patients, the elderly, and multimorbid patients, evidence suggests that rivaroxaban can be utilized as a substitute for LMWH, provided renal and hepatic function are normal and drug interactions are managed.

In summary, rivaroxaban and LMWH share a comparable foundation of clinical efficacy but differ in pharmacologic properties, safety profiles, and convenience. These differences must be weighed carefully in tailoring therapy to patient-specific needs, forming the following rationale for clinical comparisons in Section 4.

## 4 Clinical efficacy of rivaroxaban vs. LMWH

Rivaroxaban has been extensively investigated with respect to effectiveness in the prevention and treatment of venous thromboembolism (VTE) in the most challenging groups, i.e., cancer patients or following orthopedic surgery. This section pools the evidence of superior quality of real-world data and meta-analyses, and clinical trials to promote a stronger level of knowledge on the contrasts between rivaroxaban and LMWH in such scenarios.

### 4.1 Patient outcomes and effectiveness

The measures to be considered in assessing the effects of anticoagulant therapy are VTE recurrence, bleeding events, and the mortality related to any cause (Kohn et al., 2019). Several studies concluded that rivaroxaban and LMWH cannot be considered any more efficient in their effects on patients with cancer-related thromboses, as both drugs are efficient in preventing the recurrence of VTE (Wu et al., 2024a). This tradeoff between safety and efficacy is fundamental in cancer patients, as the anticoagulation treatment should mitigate the impact of thrombosis without increasing bleeding complications.

**FIGURE 3:**
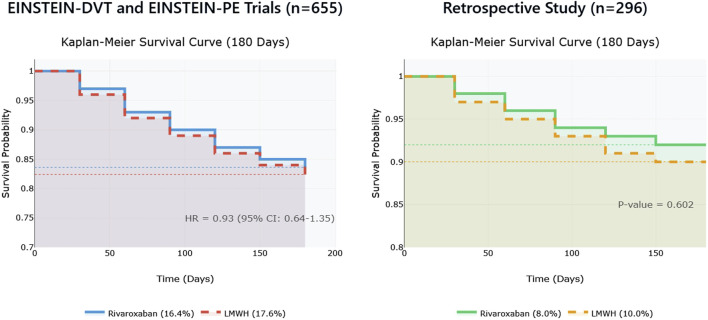
Rivaroxaban versus low-molecular-weight heparin: Mortality Analysis in Cancer Patients with Venous Thromboembolism.

Among 655 cancer patients with VTE (EINSTEIN-DVT and EINSTEIN-PE), rivaroxaban had a non-significant trend toward reduced mortality versus LMWH (16.4% vs. 17.6%; hazard ratio [HR] = 0.93, 95% CI: 0.64–1.35, p = 0.30), suggesting possible benefits in survival, although not statistically significant (Xing et al., 2019). Similarly, a retrospective cohort study of 296 cancer patients with VTE showed a trend toward lower mortality in the rivaroxaban group (8.0% vs. 10.0%, p = 0.602) (Dong et al., 2024). Though these results were insignificant, the consistent trends in studies are interesting and suggest that rivaroxaban may have a potential survival benefit in cancer patients, which needs further exploration. Figure 3 illustrates mortality in this study.

Postoperative hypercoagulability in orthopedic surgery, particularly after total hip arthroplasty, is also prevented importantly by rivaroxaban. A retrospective analysis of 110 patients proved rivaroxaban to be linked with fewer patients showing signs of postoperative day 3, 5, and 7 hypercoagulability (41.67% vs. 72%; 60% vs. 84%; 76.67% vs. 92%; p < 0.05) (Sun et al., 2020). This indicates that rivaroxaban can offer improved thromboprophylaxis, lowering the incidence of VTE and facilitating post-surgery recovery.

### 4.2 Real-world evidence in medical inpatients

Real-world data are essential in determining real-world outcomes of anticoagulants like rivaroxaban in daily clinical practice. In a SEER-Medicare study of patients with cancer and VTE, rivaroxaban was compared with LMWH for recurrent VTE, major Bleeding, and all-cause mortality. The results were that none of the differences in outcome were statistically significant: Recurrent VTE (HR = 0.91, 95% CI: 0.51–1.62), Major Bleeding (HR = 0.90, 95% CI: 0.29–2.83), All-cause mortality (HR = 0.86, 95% CI: 0.62–1.21) (Kohn et al., 2019). These findings confirm the efficacy and safety equivalence of the two drugs in real-world use, suggesting that both rivaroxaban and LMWH possess similar efficacy in preventing recurrent VTE and controlling bleeding risk. Nevertheless, rivaroxaban is preferable because it is orally administered and thus can be used for long-term use, particularly in outpatient settings.

Integrating real-life evidence into clinical decisions is essential to address patient-specific concerns such as comorbidities, drug interactions, and compliance. As highlighted by Beyer-Westendorf et al. (2016), the role of rivaroxaban in individualized anticoagulant therapy can optimize treatment among various categories of patients with a flexible and patient-centered strategy towards preventing VTE.

### 4.3 Summary table of efficacy outcomes

Relative efficacy of rivaroxaban and LMWH is discussed below in Table 2, which collects significant results from clinical trials and real-world data:

**TABLE 2:** Clinical Outcomes of Rivaroxaban vs. Low-Molecular-Weight Heparin (LMWH) in VTE Management.

Outcome	Rivaroxaban	LMWH	p-value
VTE Recurrence	Similar efficacy	Similar efficacy	Not significant
Major Bleeding	1.2%	1.5%	p > 0.05 (Deitelzweig et al., 2015)
All-Cause Mortality	Slightly lower (16.4%)	Slightly higher (17.6%)	p = 0.602 (Dong et al., 2024)
Cancer-related Thrombosis	Non-inferior to LMWH	Non-inferior to rivaroxaban	Not significant (Wu et al., 2024b)
Orthopedic Surgery Outcomes	Better hypercoagulability prevention	Worse hypercoagulability prevention	p < 0.05 (Sun et al., 2020)

Rivaroxaban is equally effective as LMWH in the prevention and treatment of VTE in most clinical scenarios, including cancer-associated thrombosis and orthopedic surgery. Although there are minor differences in bleeding risks, rivaroxaban’s oral administration is extremely convenient for patients and is non-inferior to LMWH for serious Bleeding and VTE recurrence (Kohn et al., 2019). Real-world experience also substantiates the use of rivaroxaban in daily clinical practice, with the safety and efficacy of rivaroxaban established in diverse populations. With continually more precise data from further research, the place of rivaroxaban in patient-specific, individualized anticoagulation will only grow.

### 4.4 Special population efficacy

While rivaroxaban and LMWH are effective within the general VTE population, limited data are available on their effectiveness in high-risk subgroups with renal impairment, liver disease, or elderly patients. In more recent years, these gaps have begun to be filled:

Renal Impairment: In a paper by McBane et al. (2016), patients with moderate renal impairment (CrCl 30–50 mL/min) were assessed for rivaroxaban. The results revealed that rivaroxaban was similar to LMWH for preventing recurrent VTE. However, dosing was possibly needed in patients with severe renal impairment (CrCl <30 mL/min) due to increased drug exposure (Nair et al., 2020). LMWH is still preferred in the latter group due to its more linear clearance and ability to monitor anti-Xa levels.

Liver Dysfunction: Liver disease poses a unique challenge for anticoagulation therapy. Rivaroxaban is not recommended in patients with moderate or severe impairment of the liver since drug metabolism is altered and an increased risk of bleeding cannot be excluded. However, in patients with mild liver impairment, rivaroxaban can be given cautiously. However, LMWH is generally preferred due to its consistent pharmacokinetics and good profile in patients with liver impairment (Jones et al., 2024).

Geriatric Populations: Geriatric patients (≥75 years) are at higher risk of adverse effects on bleeding. A subgroup analysis of the EINSTEIN trials showed that rivaroxaban was well tolerated in the geriatric patient group. However, careful monitoring of bleeding symptoms, especially in patients with comorbidities such as hypertension or renal failure, was necessary (Xing et al., 2019). LMWH remains first-line in this age group if renal function is compromised due to the ease with which the dose can be modified and its established safety profile. Further research, primarily randomized controlled trials in such subgroups, is necessary to standardize these populations’ management further.

## 5 Risk factors and patient selection for anticoagulation therapy

Optimum anticoagulant treatment with agents such as rivaroxaban and low-molecular-weight heparin (LMWH) must consider patient-specific parameters to prevent venous thromboembolism (VTE). Individualization of treatment with references to contraindications and patient needs should be offered to achieve the best effect of treatment (Gorog et al., 2022). This section summarises the description of high-risk patient group identification, individualisation of therapy principles, and the absolute contraindications to anticoagulation therapy.

### 5.1 Identification of high-risk patient populations

Correctly identifying high-risk patients guarantees maximum benefit gain in the anticoagulation therapy compared to the side effects of use. Patients at high risk include those with a history of pulmonary or thrombotic disease, cancer, atrial fibrillation, and critically ill patients. Certain conditions and groups of patients have to be assessed critically:

Cancer Patients: Cancer significantly increases the risk of VTE. Cancer-related thrombosis is common, particularly in chemotherapy patients, who need personalized anticoagulation therapy (Rus et al., 2024).

Atrial Fibrillation: Anticoagulation risk stratification in atrial fibrillation is typically done with the CHA2DS2-VASc score to estimate thromboembolic risk, and the HAS-BLED score to assess bleeding risk (Gorog et al., 2022).

Renal Impairment: Renal dysfunction patients have an increased risk of bleeding and of inadequate anticoagulation. Dosage adjustment is necessary to achieve a therapeutic effect without diminished safety.

Older Patients: Elderly patients, especially those over the age of 75 years, are at higher risk of bleeding due to altered pharmacokinetics with age and comorbidities.

By early recognition of such high-risk populations, clinicians can intervene early and appropriately to optimize patient outcomes.

### 5.2 Individualizing therapy to a Patient’s need

Individualized anticoagulation treatment is successful. Rivaroxaban or LMWH can be chosen depending on several factors, including renal function, comorbid conditions, compliance, and patient preference. These guide the selection of treatment to provide the best for the patient.

Rivaroxaban: Rivaroxaban is a direct Factor Xa inhibitor taken orally with predictable pharmacokinetics, so it is best suited for outpatient settings. Oral intake enhances patient compliance and is specifically helpful in patients who do not like injections. In addition, it does not need regular monitoring and has fewer food interactions, enhancing its long-term treatment use (Tanaka-Esposito and Chung, 2015).

LMWH: Inpatient treatment or, in the event of emergency anticoagulation, is the most frequent use of LMWH, as it acts immediately with a therapeutic effect by subcutaneous injection. LMWH can be conveniently adjusted based on anti-Xa monitoring, which is helpful in renal failure or other special needs (Rus et al., 2024). Surgical patients benefit from the adjustable dose of LMWH and its established efficacy as the first-choice drug in short-term therapy.

Both medications require careful dose modification in patients with renal impairment. For instance, rivaroxaban is contraindicated in patients with severe renal impairment (CrCl <15 mL/min). At the same time, LMWH requires dose adjustment in moderate renal impairment to avoid drug accumulation and bleeding to ensure its efficacy (Sherrard et al., 2016). A tailored treatment considers patient values, lifestyle, and compliance with therapy. Collaborating with patients and families in the decision process for treatment, which is aligned with personal preference, for instance, an oral medication for ease or an injectable drug for improved acute control, will significantly impact the outcome.

### 5.3 Contraindications and cautions

Safe anticoagulant therapy relies on the establishment of absolute and relative contraindications. These contraindications must be carefully assessed before initiating treatment to serve as a protective measure for the patient and to prevent harm.

#### 5.3.1 Absolute contraindications

Active Major Bleeding: Any significant hemorrhage, such as gastrointestinal or intracranial.

Recent Hemorrhagic Stroke: Patients with a history of hemorrhagic stroke should not receive anticoagulation therapy since rebleeding can happen.

Hypersensitivity: Any established hypersensitivity to the drug or to any part of it constitutes an absolute contraindication.

#### 5.3.2 Relative contraindications

Recent Surgery: Patients who have undergone significant surgery, especially central nervous system or eye surgery, require anticoagulant therapy with caution.

Peptic Ulcers: Patients with ongoing peptic ulcer disease or a history of gastrointestinal hemorrhage should be monitored closely.

Liver dysfunction: Patients with moderate to severe hepatic impairment should avoid using rivaroxaban, because abnormal liver function affects the clearance and metabolism of these agents.

Transient Ischemic Stroke: This situation requires a middle-path approach, with proper monitoring of bleeding complications during anticoagulant therapy.

In patients with high-risk bleeding, such as the elderly, especially over 75 years old, renal insufficiency patients, or cancer patients, anticoagulant therapy should be administered cautiously. If there are risk factors such as uncontrolled hypertension and concomitant use of non-steroidal anti-inflammatory drugs, they should be modified as far as possible (Tsujisaka et al., 2023). In addition to these factors, polypharmacy must be approached with care in older people to avoid drug-drug interactions. For example, rivaroxaban is metabolized and transported by P-glycoprotein and CYP3A4. Thus, its concentrations in the blood can be increased by CYP3A4 inhibitors or inhibitors of P-gp, thus increasing bleeding risk (Ton et al., 2023). Summary of Contraindications and Cautions in Table 3.

**TABLE 3:** Summary of contraindications and cautions.

Contraindication	Rivaroxaban	LMWH
Active Major Bleeding	Absolute	Absolute
Recent Hemorrhagic Stroke	Absolute	Absolute
Hypersensitivity to Drug	Absolute	Absolute
Recent Surgery	Relative	Relative
Peptic Ulcers	Relative	Relative
Liver Impairment	Avoid in moderate to severe hepatic	No adjustment required
Renal Impairment	Avoid in CrCl <15 mL/min	Adjust dose in CrCl <30 mL/min
Polypharmacy	Caution (CYP3A4/P-gp interactions)	fewer interactions

### 5.4 Special population treatment tailoring

For high-risk populations such as renal insufficiency, liver disease, and the elderly, anticoagulation therapy must be individually tailored not to induce unwanted effects but to maximize effectiveness.

Renal Impairment: Patients with mild to moderate renal impairment can be treated with rivaroxaban with dose reduction and appropriate monitoring, especially in those with a CrCl of 30–50 mL/min. However, LMWH is a better treatment choice in severe renal impairment patients (CrCl <30 mL/min) due to the modifiable dosing and more stable anticoagulant effect, Monitoring anti-Xa factor (Wang et al., 2023).

Hepatic impairment: Rivaroxaban is acceptable for patients with mild hepatic impairment. Nevertheless, LMWH is usually favored because it can be administered on a patient-specific basis regarding renal function and is relatively predictable in its off-site impact. Hepatic moderating to severe impairment is contraindicated for rivaroxaban (Khorana et al., 2017). LMWH is preferable in this kind of patient, particularly in VTE, where the risk of bleeding should not be enhanced.

Older Populations: Older patients are more at risk of having bleeding episodes. In the elderly, rivaroxaban therapy will require close monitoring (based on renal clearance and dose reduction based on weight). The HAS-BLED score can assist in assessing the risk of bleeding (Heisen et al., 2017). In the elderly (75 years and upwards) with increased bleeding risk, LMWH is preferably indicated, especially in cases of issues of medication compliance or when there are also some severe comorbidities.

## 6 Economic considerations and healthcare impacts

The cost analysis of drugs such as rivaroxaban and low-molecular-weight heparin (LMWH) is important in establishing their usefulness to healthcare systems. Venous thromboembolism (VTE) is a disease that significantly affects the healthcare industry of different countries and is managed by anticoagulants (Sherrard et al., 2016). Cost-effectiveness is the primary concern of clinicians, policymakers, and medical practitioners when purchasing drugs such as rivaroxaban; the issue of rising healthcare spending necessitates such an interest. In this section, the cost-utility of rivaroxaban versus LMWH in VTE, Quality-Adjusted Life-Year (QALY), new evidence, and the cost of rivaroxaban in practice shall be untangled. QALY is a standardized measure of disease burden that combines both the quantity and quality of life generated by a healthcare intervention. It is the primary metric used in health economic evaluations to assess the value and cost-effectiveness of medical treatments, procedures, and public health programs.

### 6.1 Cost-benefit analysis of rivaroxaban vs. LMWH

The advent of oral anticoagulant rivaroxaban has resulted in several papers evaluating its cost-effectiveness with conventional therapy, i.e., LMWH. The issue of treating VTE using anticoagulant medicine has an enormous economic impact, as the burden of this investment alone adds significant value to medical care across the globe (Khorana et al., 2017). The ease of administration of rivaroxaban is due to the solid nature of this medication, which does not require extraordinary storage conditions or intravenous administration. This suffices to decrease the use of medical resources and render rivaroxaban cost-effective in some cases. In one study of patients facing surgery (total hip replacement), rivaroxaban prophylaxis for a total of 35 days was found to be based on SEK 29,400 per QALY. The other option, 14 days of dalteparin sodium and/or enoxaparin use amounts to SEK 35,400/QALY (Heisen et al., 2017). This will mean that, though a higher dose clinically, rivaroxaban may be more economical when considering the long-term effectiveness, since this drug is a tablet and does not need much monitoring.

**FIGURE 4:**
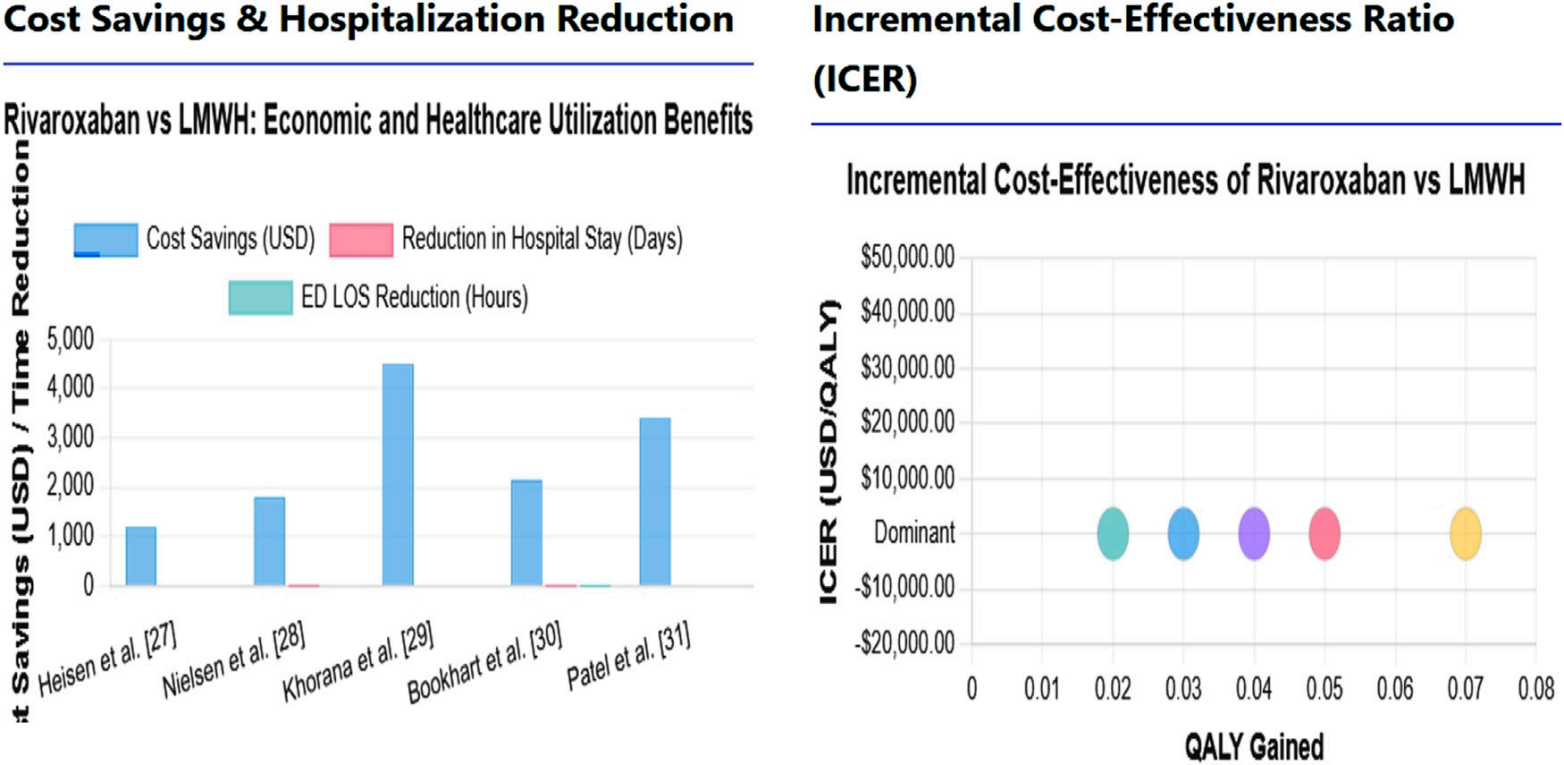
Cost-Effectiveness Analysis: Rivaroxaban vs. Low-Molecular-Weight Heparin for Venous Thromboembolism.

According to the research conducted by Nielsen et al. (2019) in the US, the sum of all medical costs of the rivaroxaban group was about 1800 dollars lower than that of LMWH. The optimal level of cost savings was achieved in cases of cancer-induced VTE, and savings were observed at an impressive figure of 4,500 dollars per patient compared to LMWH therapy with rivaroxaban (Khorana et al., 2017). These results demonstrate the economic advantage of rivaroxaban in patients with cancerous thrombosis, since such patients require a prolonged anticoagulant establishment. The redemption cost of rivaroxaban as a drug to treat pulmonary embolism in cases of emergencies was overwhelming. The patients taking rivaroxaban were also admitted and spent a reduced time in the emergency unit, resulting in 2150-dollar $2,150 savings in the LMWH-Warfarin combination (Bookhart et al., 2013). Patel et al. (2015) have found that decreased monitoring needs of rivaroxaban are less expensive, and the average total cost-saving per patient was 3,400 dollars due to the decrease in consumption of healthcare resources (Figure 4).

### 6.2 Hospital workflows and resource use impacts

Rivaroxaban use in oral delivery is highly disruptive to the hospital workflow due to transferring VTE care to the outpatient setting. This is in contrast with subcutaneous therapy with LMWH, which complicates the logistics of administering anticoagulants due to the need to supply staff administration resources to hospitals and is a challenge to manage, seeing that the complex process of administering anticoagulants is through an intracutaneous process (Heisen et al., 2017). The simplicity of the therapy negates the load on health personnel’s resources, allowing them to spare time on other important activities. In addition, rivaroxaban is pharmacokinetically stable, and there is no food effect, decreasing the number of routine coagulation tests typically required with LMWH (Bookhart et al., 2013). This eliminates the pressures of laboratory space and requires time and cost savings on repeat testing. Second, there is a decreased requirement for repeat visits with oral dosing, hence there is also a minimised traffic on outpatient clinics and consequent emergency and outpatient expenditure. This statement was confirmed in a study conducted by Merli et al. (2016) on the spending pattern of rivaroxaban and LMWH-Warfarin patients; rivaroxaban patients had 48% lower spending on emergencies and 50% lower spending on outpatients, and an overall average saving of $289 per patient per month. Such savings could be game-changing in the healthcare environment where VTE costs are minimized, particularly in low-income hospitals.

### 6.3 Long-term economic outcomes

The long-term assessments of rivaroxaban still sustain the fact that the drug is cost-effective, particularly with fewer readmissions and fewer complications. A conceptual commercial health plan using rivaroxaban forecasted a 5 percent cut in inpatient days, which can indicate complication reduction of VTE treatment. It resulted in substantial savings (Patel et al., 2015). The central aspect that makes rivaroxaban very cost-effective in the long term is its dependable clinical outcomes and higher levels of patient compliance. Enhanced adherence to anticoagulation care also increases typical health outcomes, including diminished VTE recurrence and sequelae, further minimizing medical costs (Smith et al., 2018). Besides, compliance has been found to prevent readmission, resulting in a reduced length of stay and a reduction of the overall expenditure of healthcare costs (Bookhart et al., 2013). These long-term returns indicate that even though rivaroxaban is more expensive upfront, it can potentially reduce the overall healthcare expenditure due to its practical benefits of long-term management and patient outcomes.

## 7 Future directions for research and clinical practice

The sphere of anticoagulation therapy is transforming based on enhancing drug development, personalized approaches, and reversal agents. One of the significant reforms evident in VTE treatment is the introduction of direct oral anticoagulants (DOACs) like rivaroxaban, a safe and convenient addition (Yamashita et al., 2022). There are important trends that will guide anticoagulant therapy based on emerging evidence.

### 7.1 Trends and innovations emerging in anticoagulant therapy

The future has to be defined by continuous innovations, particularly regarding the potential for improved efficacy, enhanced safety, and minimized side effects. Among the most interesting tendencies, the enhanced use of the DOACs, including rivaroxaban, exposes consequences immeasurably greater compared to the usual treatment presented in warfarin (Lo et al., 2022). The oral administration of DOACs has reliable pharmacokinetics and low dietary requirements, and they do not require frequent coagulation monitoring. These drugs are revolutionizing the course of treatment of the conditions of atrial and VTE fibrillation and long-term treatment regimens (Yamashita et al., 2022). Individualized anticoagulation care in accordance with genetic susceptibility and burden of disease is among the breakthroughs in the sector. The pharmacogenetic field helps every individual make the wisest selection of anticoagulant medication. New studies focusing on the genetic variation of cytochrome P450 enzymes, which increase or decrease the efficacy of DOACs, have been in development (Kotalczyk et al., 2021). Abelacimab is a fully human monoclonal antibody. In a Phase II clinical trial assessing VTE prevention after total knee arthroplasty, the 75 mg abelacimab group (5%; 95% CI: −26.0 to −7.6, p < 0.001) and the 150 mg group (4%; 95% CI: −26.7 to −8.8, p < 0.001) demonstrated significantly superior efficacy compared to enoxaparin (22%) (Verhamme P et al., 2021). This indicates that postoperative inhibition of factor XI is an effective and safe anticoagulation strategy that has the potential to achieve the goal of ‘antithrombosis without causing bleeding’.

### 7.2 Reversal agent development

The other area where rapid advancements are being achieved is in the preparation of reversal agents for the DOACs, e.g., for rivaroxaban. The change in the effect of DOACs is significant; such factors include reversal agents, such as asexanet alfa (Factor Xa agents) and idarucizumab (Dabigatran) (Milling et al., 2023). This is required in the acute setting, where it is in the interest of antidoting against anticoagulation and expediently accomplishing this. This will make the agents more available and affordable, thus widening the antithrombotic safety balance of DOACs and, accordingly, increasing their applicability in the real clinical environment (Smith et al., 2018).

### 7.3 Gaps in current knowledge and implications for future studies

Despite these advances, there are still gaps in the knowledge related to research on anticoagulation treatment. Findings regarding the safety and efficacy of DOACs in patients with end-stage renal and liver failure, pregnant women, and those with a history of active cancer are of utmost interest in future studies (Kotalczyk et al., 2021). Guidance documents are pieces of general advice, but in high-risk populations, clinical trial information is poor (Wang et al., 2023). Moreover, cross-reaction of other drugs, especially with anticoagulants and in combination therapy (dual/triple therapy; patients taking antiplatelet and anticoagulants), needs additional studies (Erkan et al., 2014). The interactions of DOACs with coadministered drugs among patients with acute coronary syndrome or undergoing percutaneous coronary intervention will be simplified to optimize the regimen and to minimize bleeding (Lo et al., 2022).

Finally, research into resistance and failure of DOAC, potentially caused by drug-drug interaction, drug absorption issues, or non-compliance, is critical. Such research will ensure that patients receive the optimum and safest treatment, reducing repeated occurrences of VTE despite proper anticoagulation (Jones et al., 2024).

### 7.4 Clinical practice patterns and clinical guidelines recommendations

With the increasing advancements in the field of medicine, clinical guidelines have to be updated with the latest evidence, especially pharmacogenomic information and novel treatment modalities. Clinical practitioners must be educated on the nuances of anticoagulant selection and patient-specific factors, thereby improving the care of patients. Implementing decision-support tools within electronic health records will also enable clinicians to adhere better to guidelines and make informed, patient-centered decisions (Butler et al., 2022). Also, closing gaps in access to anticoagulant therapy will give all patients equal treatment regardless of socioeconomic status.

## 8 Conclusion

This article highlights the effectiveness of both rivaroxaban and LMWH in preventing VTE in hospitalized patients. Oral dosing of rivaroxaban has distinct advantages, such as easier use, reduced nursing time, and simpler transition to outpatient management, without sacrificing efficacy, and with a similar risk of major bleeding compared to LMWH. Choice of anticoagulant should be individualized according to renal function, bleeding risk, and cancer status. In situations where the administration of anticoagulant is rapid or oral agents are contraindicated, LMWH is the preferred drug. Forward, continued research is needed to address special populations’ safety issues, increase reversal agent availability, Development of new anticoagulants, and integrate pharmacogenomics into clinical practice guidelines. Effective integration of these advancements will educate clinicians to deliver more individualized, less costly care. By optimizing clinical practices and policies, healthcare systems will be better positioned to address the diverse needs of patients with VTE, enhance patient outcomes, and reduce healthcare costs.

## Forward-looking statement disclaimer

This conclusion contains forward-looking statements based on current evidence and expectations regarding the future integration of anticoagulant therapies. These statements involve known and unknown risks, uncertainties, and other factors that may cause actual results, performance, or achievements in clinical practice to differ materially from those expressed or implied. These factors include, but are not limited to, the results of ongoing and future clinical trials, regulatory approvals, availability of reversal agents, evolution of clinical guidelines, and practical challenges in healthcare implementation. We undertake no obligation to update any forward-looking statements to reflect events or circumstances after the date of this document.
